# Memory Networks in Tinnitus: A Functional Brain Image Study

**DOI:** 10.1371/journal.pone.0087839

**Published:** 2014-02-06

**Authors:** Maura Regina Laureano, Ektor Tsuneo Onishi, Rodrigo Affonseca Bressan, Mario Luiz Vieira Castiglioni, Ilza Rosa Batista, Marilia Alves Reis, Michele Vargas Garcia, Adriana Neves de Andrade, Roberta Ribeiro de Almeida, Griselda J. Garrido, Andrea Parolin Jackowski

**Affiliations:** 1 Laboratório Interdisciplinar de Neurociências Clínicas (LiNC), Departamento de Psiquiatria, Universidade Federal de São Paulo, São Paulo, Brasil; 2 Departamento de Otorrinolaringologia e Cirurgia de Cabeça e Pescoço, Universidade Federal de São Paulo, São Paulo, Brasil; 3 Seção de Medicina Nuclear, Departamento de Radiologia, Universidade Federal de São Paulo, São Paulo, Brasil; 4 Instituto do Cérebro – Hospital Israelita Albert Einstein, São Paulo, Brasil; 5 Departamento de Fonoaudiologia, Universidade Federal de São Paulo, São Paulo, Brasil; 6 Western Australian Centre for Health and Ageing, Centre for Medical Research, University of Western Australia, Perth, Australia; University of Regensburg, Germany

## Abstract

Tinnitus is characterized by the perception of sound in the absence of an external auditory stimulus. The network connectivity of auditory and non-auditory brain structures associated with emotion, memory and attention are functionally altered in debilitating tinnitus. Current studies suggest that tinnitus results from neuroplastic changes in the frontal and limbic temporal regions. The objective of this study was to use Single-Photon Emission Computed Tomography (SPECT) to evaluate changes in the cerebral blood flow in tinnitus patients with normal hearing compared with healthy controls. Methods: Twenty tinnitus patients with normal hearing and 17 healthy controls, matched for sex, age and years of education, were subjected to Single Photon Emission Computed Tomography using the radiotracer ethylenedicysteine diethyl ester, labeled with Technetium 99 m (99 mTc-ECD SPECT). The severity of tinnitus was assessed using the “Tinnitus Handicap Inventory” (THI). The images were processed and analyzed using “Statistical Parametric Mapping” (SPM8). Results: A significant increase in cerebral perfusion in the left parahippocampal gyrus (pFWE <0.05) was observed in patients with tinnitus compared with healthy controls. The average total THI score was 50.8+18.24, classified as moderate tinnitus. Conclusion: It was possible to identify significant changes in the limbic system of the brain perfusion in tinnitus patients with normal hearing, suggesting that central mechanisms, not specific to the auditory pathway, are involved in the pathophysiology of symptoms, even in the absence of clinically diagnosed peripheral changes.

## Introduction

Tinnitus is an auditory phantom sensation, described as ringing or buzzing in one or both ears, in the absence of an external auditory stimulus. The prevalence of chronic tinnitus varies from 5–15%, and 1–3% of these cases severely affect the quality of life, resulting in anxiety and emotional disorders, sleep disturbance and work impairment [1].

Over 20 years, it has been suggested that tinnitus reflects peripheral pathology, primarily cochlear dysfunction or auditory nerve damage [2]. Recently, imaging and electrophysiological methods have suggested the involvement of central mechanisms in generation and perception of tinnitus symptoms. The central origin of tinnitus-related activity has replaced the peripheral hypothesis [3]–[6]. Increased spontaneous neural activity, synchrony or reorganized tonotopic maps have been proposed as neural substrates of tinnitus [7].

Groundbreaking imaging studies have shown structural and functional brain changes associated with tinnitus [8], although the exact location remains controversial [9]. Abnormal activity might be interpreted and perceived as tinnitus in higher cortical centers and areas associated with auditory and emotional processing [3]. Recent studies using Magnetic Resonance Imaging (MRI), Positron-Emission Tomography (PET), and Single-Photon Emission Computed Tomography (SPECT) have demonstrated the involvement of non-auditory brain areas in a global network that encodes subjective tinnitus. These techniques provide evidence that structural and functional changes in temporal, prefrontal and limbic structures are the basis for tinnitus symptomatology [8], [10]. Others studies using electrophysiological tests have demonstrated abnormal connectivity between auditory and non-auditory networks [11], and several concepts have been proposed to explain this involvement [12]–[13].

Conscious sound processing in auditory centers and the association of the signal with unpleasantness and distress might be responsible for the interpretation of the aberrant auditory signal as troublesome tinnitus [14]. These processes are influenced by the memory, attention and emotional state of the patient. It has been proposed that auditory centers play a major role in the generation of tinnitus-related activity, although tinnitus might be initially triggered through peripheral auditory deficits, even with a normal audiogram [5]. Schaette and McAlpine (2011) demonstrated that even in absence of elevated hearing thresholds, a fraction of auditory nerve fibers no longer respond to sound, and tinnitus could result from a homeostatic response of the central auditory system to reduced auditory nerve input [15]. Taken together, these observations suggest that tinnitus results from peripheral injury, the reorganization of central auditory pathways and changes in the encoding of the emotional content of sensory experiences in portions of limbic system.

Sataloff et al. (1996) demonstrated the diagnostic benefits of SPECT in assessing neurotological complaints. These researchers observed that among other techniques, including MRI, computed tomography (CT) and electroencephalography (EEG), SPECT is a more sensitive method for the analysis of neurotological conditions, such as dizziness, hearing loss and tinnitus. SPECT abnormalities were observed in 92% of tinnitus patients, while only 44% MRI and 22% computed tomography (CT) scan abnormalities were observed among these same patients [16]. Few studies have explored the use of SPECT for the investigation of dynamic and metabolic brain changes in tinnitus [16].

The purpose of the present study was to characterize the brain activity in patients with tinnitus and normal hearing using SPECT.

## Methodology

### Subjects

Twenty tinnitus patients and 17 control subjects, matched by age, sex, years of education and hearing, were enrolled in this study. The study participants were recruited among patients seeking treatment at the Tinnitus Center of the Federal University of São Paulo and through the website of the same institution.

The inclusion criteria were continuous uni- or bilateral tinnitus for longer than 3 months, between 18 and 60 years of age and normal hearing in both ears. All patients underwent complete otological and audiological examinations, including pure tone audiometry, tympanometry, stapedius reflex tests and otoscopy. Pure tone audiometry was performed using a clinical audiometer with 8 different octave frequencies (0.25, 0.5, 1, 2, 3, 4, 6 and 8 kHz). The exclusion criteria included thresholds >25 dB hearing level in any frequency, neurologic or psychiatric disorders, particularly epilepsy, migraine, or schizophrenia, pregnancy, and breastfeeding women. The Portuguese version [17] of the Tinnitus Handicap Inventory (THI) [18] was used to assess tinnitus severity, classified as none (0–16), mild (18–36), moderate (38–56) or severe (58–100) [19].

### Ethics Statement

The local research ethics committee, “Comitê de Ética em Pesquisa da Universidade Federal de São Paulo/Hospital São Paulo”, approved this study (protocol 2094/09). All participants provided written informed consent for clinical investigation and subsequent analysis after a comprehensive explanation of the procedures.

### Assessment of Regional Brain Perfusion Using 99mTc-ECD SPECT in Tinnitus

SPECT was performed with an ethyl cysteinate dimer labeled with technetium-99 m (99mTc-ECD) using a double-head gamma camera (GE® Infinia - USA) equipped with a pair of low-energy fan-beam collimators.

The 99mTc-ECD was prepared from a lyophilized kit (IPEN ECD®) by adding from 3700 MBq/2 mL (100 mCi) freshly eluted 99mTc-pertechnetate from a 99Mo/99mTc generator (IPEN-Tec®). Before labeling, the 99mTc must be freshly eluted (<2 hours) and the content of 99Mo must be determined. The radiochemical purity of the 99mTc-ECD (>90%) was determined through solvent extraction (3 mL of a 0.9% saline solution and 3 mL of chloroform).

All subjects rested with closed eyes in a dark quiet room for 30 min, followed by the intravenous injection of 25–30 mCi (925–1110 MBq) 99mTc-ECD and an additional 30 min of rest. Acquisition was achieved through 128 projections on a 128×128 matrix, with 40,000 counts per projection. The images were reconstructed as transaxial slices parallel to the orbitomeatal line, using filtered back projection with a Butterworth filter (cut-off frequency 0.48 cycles/pixel and order 10) and Chang’s method for attenuation correction.

### Data Analysis

For the analysis of the SPECT data, the reconstructed ECD images were exported to an NIfTI file format and processed using Statistical Parametric Mapping 8 [20] of the Wellcome Department of Imaging and Neuroscience, London (http://www.fil.ion.ucl.ac.uk/spm) using Matlab® 7.0 (Mathworks®, Sherborn, MA, USA). Morbelli et al (2008) showed that using the SPM8 standard HMPAO SPECT template for the spatial normalization of ECD SPECT images yields incorrect results [21]. To more closely match the template to the sample under investigation, the original images were linearly matched to a smoothed FWHM = 8 mm Gaussian kernel version of the MNI ECD SPECT template downloaded from the website according to Morbelli et al (2008) [21]. The resulting images were averaged and subsequently matched to a smoothed FWHM = 8 mm Gaussian kernel to create the study template. In a second phase, the original images were non-linearly deformed to the customized template yielding 2 mm×2 mm×2 mm images matched to a smoothed FWHM = 8 mm Gaussian kernel to account for interindividual brain anatomy differences and increase the signal-to-noise ratio. The images were globally normalized for signal intensity using proportional scaling to remove the confounding effects of global CBF changes, with a threshold of 0.8, and include only those voxels with intensities exceeded 80% of the global mean. Furthermore, a gray matter binary mask was used as an explicit mask to exclude white matter voxels from the statistical analysis.

### Statistical Analysis

The demographic and baseline clinical characteristics between groups were compared using unpaired t-tests. We performed a descriptive analysis of the THI, considering emotional, functional, catastrophic, and final scores.

Voxel-based comparisons of the regional tracer uptake values between tinnitus patients and controls were performed using a two-sample t-test. The SPM results were displayed at p<0.05 uncorrected, with a cluster extent threshold = 50 voxels. The areas were identified using MRIcron (http://www.nitrc.org/projects/mricron) and xjview (http://www.alivelearn.net/xjview8).

The ROIs for SVC correction were created using MRIcron (http://www.mccauslandcenter.sc.edu/mricro/mricron/) version 6.6.2013 after one expansion of the temporal and limbic system regions extracted from the Automatic Anatomical Labeling (AAL) template [22]. After SVC correction, the results that survived at pFWE (Family-wise error) <0.05 at the peak level were accepted. The post hoc analyses showed that the pattern of the findings remained virtually unaltered after covarying for age and gender.

## Results

No differences were observed between control individuals and tinnitus patients regarding age (42.95+9.03 years versus 41.41+9.98 years, respectively; p = 0.613), gender (6 males and 14 females versus 6 males and 11 females, respectively; p = 0.985), or years of education (11.2+3.87 versus 11.41+3.78, respectively; p = 0.860). The total mean score for disease severity was 50.8+18.24. The demographic and THI test scores (total, physical, emotional, and catastrophic domains) are presented in Table 1.

**Table 1:** Demographic and clinical characteristics of the study participants.

		TI	Controls
Gender	Male	06	06
	Female	14	11
Age (Mean+SD)		42.95 (9.03)	41.41 (9.98)
Years of education (Mean+SD)		11.2 (3.87)	11.41 (3.78)
Pure-tone average (0.25–8 kHz)			
Mean right (+SD)		13.4 (5.65)	10.63 (4.28)
Mean left (+SD)		14.24 (4.92)	10.53 (3.86)
THI total (Mean+SD)		50.8 (18.2)	N/A
THI emotional (Mean+SD)		18.2 (7.9)	N/A
THI physical (Mean+SD)		20.4 (9.39)	N/A
THI catastrophic (Mean+SD)		12.2 (3.94)	N/A
TI laterality	Right	01	N/A
	Left	03	N/A
	Bilateral	13	N/A
	Head	03	N/A

THI, Tinnitus Handicap Inventory; TI, Tinnitus.

**Figure 1:**
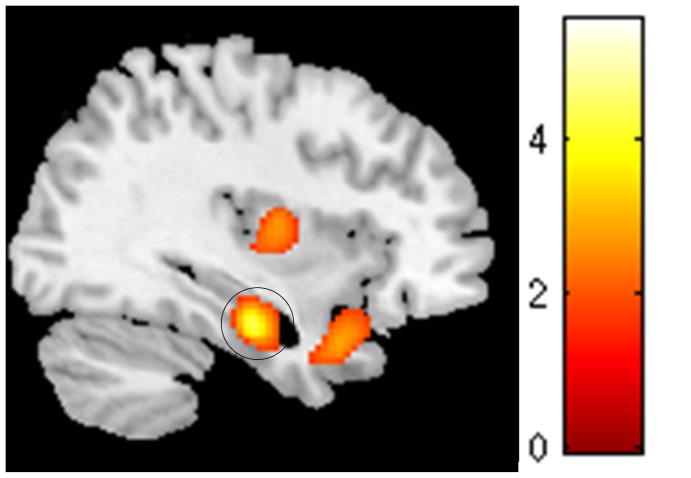
rCBF increase (pFWE <0.05 after SVC) in the left parahippocampal gyrus in tinnitus patients compared with control subjects.

A comparison of the SPECT images revealed a significant increase in the metabolic activation of the left parahippocampal gyrus (pFWE<0.05, Table 2 and Figure 1) in patients compared with the controls. The stability of the results after covarying for age and gender suggested that the observed differences are associated with the effect of interest.

**Table 2:** Regional cerebral blood flow increase in tinnitus patients compared with control subjects.

Brain regions	MNI Coordinates of the peak voxel			Cluster size		pFWE after SVC
Increase	x	y	z	K_E_	t	
Parahippocampal_L	−26	−2	−30	1322	5.02	0.021

FWE, Family-wise error; KE, number of voxels per cluster;

No unexpected findings survived at pFWE<0.005 at voxel level.

## Discussion

Using 99mTc-ECD SPECT, the results obtained in the present study showed differences in the brain activation patterns in tinnitus patients with clinically normal hearing compared with healthy controls. The availability of an objective technique, whereby the location, extent and magnitude of neural activity can be measured, might greatly contribute to the understanding of tinnitus pathophysiology, as these processes are subjective and cannot be measured using laboratory tests. However, the use of SPECT to characterize tinnitus pathophysiology remains poorly described. There have only been 11 studies using SPECT for the investigation of these symptoms, with varying methodologies [3]; [14]–[15]; [23]–[30].

The functional brain abnormalities observed in the present study were evaluated in the presence of normal audiogram results, using carefully matched groups with respect to gender, age, years of education and hearing, to minimize or avoid the interference of these factors in the results shown. To date, only 3 SPECT studies have compared healthy controls with tinnitus patients; however, none of these studies matched the control group for the degree of hearing loss. Mahmoudiam et al (2012) examined 122 patients with subjective idiopathic tinnitus, and only 9 healthy controls were included in this study, and the hearing thresholds were not considered [30]. Farhadi et al (2010) also compared tinnitus patients with hearing loss to healthy controls, without pairing according to hearing thresholds [14]. Gardner et al (2002) compared depressed patients with and without tinnitus, but the sample of tinnitus patients only contained 3 individuals with normal hearing bilaterally, and these studies were not performed using healthy controls without depression [3].

It has recently been suggested that tinnitus without hearing loss does not necessarily indicate the absence of deafferentation [5]; [15]; [31]–[32]. It is possible that peripheral damage, particularly at high frequencies, could be present, but not detected through routine audiometry [32]. Even if patients with apparently normal audiograms show ‘hidden hearing loss’ due to damage to auditory nerve fibers [15], the control of the audiological symptoms reduces the difference between patients and controls and is a strategy that produces more consistent and reliable results associated with tinnitus. The stability of our results after covarying for age and gender suggests that the observed differences are directly associated with tinnitus.

Recent evidence on the neurobiology of tinnitus suggests that although auditory system dysfunction is necessary for tinnitus to occur, the neural changes in the presence of these symptoms are not only limited to the auditory pathway [3]. Studies have proposed that the limbic system might play a role in modulating or perpetuating tinnitus [33], and interactions between the limbic corticostriatal network and the primary auditory cortex might be the key to understanding chronic tinnitus. This limbic activation has been interpreted as a reflection of the emotional reaction of tinnitus patients to tinnitus sounds [34]. However, the precise involvement of the limbic system in tinnitus has yet to be elucidated [9].

Thus, the results obtained in the present study reinforce the current concepts. The increased cerebral perfusion in tinnitus patients was observed in the left parahippocampal gyrus, located in the medial temporal lobe, which is part of the limbic system and also considered as part of the neural circuitry associated with tinnitus [35].

It has been proposed that a fundamental function of parahippocampal structures is the establishment of auditory memory for tinnitus [23]. The parahippocampal gyrus is involved in the maintenance of tinnitus, preventing the modification or elimination of hippocampal memory, thereby avoiding habituation [36]–[37]. MRI studies have previously described the involvement of the parahippocampal region in the judgment of inharmonious and unpleasant music, showing the activation of this region in response to aversive stimuli [38]. It has been postulated that due to the strong reciprocal connection between the parahippocampal gyrus and amygdala, these structures function as components of the neural system that help the body to cautiously explore and protect itself from harmful experiences [39]. Joos et al (2012) concluded that due to parahippocampal connectivity with the anterior cingulate and orbitofrontal cortices, the parahippocampal gyrus could be the decisive link between the network associated with tinnitus and attentional-emotional neural circuitry, underlying emotional disturbances associated with the symptoms [35].

Other structures of the paralimbic network associated with the amygdala, such as the nucleus accumbens of ventral striatum (NAc) and prefrontal cortex ventromedial (vmPFC) have been implicated in the assessment of emotional sound content. Rauschecker et al (2010) recently proposed a model of tinnitus pathophysiology, which suggests that efferents are involved in the cancellation of the tinnitus signal in the thalamus, and although tinnitus signals might initially be generated in parts of the auditory system, the limbic structures are compromised, the cancellation of tinnitus signals in the thalamus is disrupted and does not prevent the tinnitus signal from reaching the auditory cortex, leading to ongoing reorganization and chronic tinnitus [40]. Recent evidence that the NAc and vmPFC do indeed differ in the brains of individuals with tinnitus support the current hypothesis [8].

We speculate that the failure to detect significant differences in the cerebral perfusion of the superior and medial temporal gyrus and frontal areas, as demonstrated in previous studies [4]; [14]; [28], might reflect the observation of clinically normal audiograms. Although there might be bias towards the periphery auditory fibers in the presence of normal audiograms, this unbalance may not be sufficient to produce a significant change in cerebral blood flow in these regions, consistent with the view that the cortical network is involved in the conscious perception of tinnitus and the emotional symptoms that accompany this condition.

In the present study, we identified a change in cerebral perfusion, which was not specific to the auditory pathway in patients with tinnitus and normal hearing, suggesting the involvement of central mechanisms in the pathophysiology of symptoms, even in the presence of clinically normal hearing.
